# Neutralization of SARS-CoV-2 BQ.1.1, CH.1.1, and XBB.1.5 by breakthrough infection sera from previous and recent waves in China

**DOI:** 10.1038/s41421-023-00569-5

**Published:** 2023-06-27

**Authors:** Xun Wang, Shuai Jiang, Shujun Jiang, Xiangnan Li, Jingwen Ai, Ke Lin, Shiyun Lv, Shixuan Zhang, Minghui Li, Jixi Li, Lili Dai, Zixin Hu, Wenhong Zhang, Yanliang Zhang, Pengfei Wang

**Affiliations:** 1 Shanghai Pudong Hospital, Fudan University Pudong Medical Center, Shanghai Institute of Infectious Disease and Biosecurity, State Key Laboratory of Genetic Engineering, MOE Engineering Research Center of Gene Technology, School of Life Sciences, Fudan University, Shanghai, China; 2 Shanghai Huashen Institute of Microbes and Infections, Shanghai, China; 3 Department of General Surgery, Shanghai Pudong Hospital, Fudan University Pudong Medical Center, Shanghai Key Laboratory of Vascular Lesions Regulation and Remodeling, Shanghai, China; 4 Department of Infectious Diseases, Nanjing Hospital of Chinese Medicine Affiliated to Nanjing University of Chinese Medicine, Nanjing, Jiangsu China; 5 Nanjing Research Center for Infectious Diseases of Integrated Traditional Chinese and Western Medicine, Nanjing, Jiangsu China; 6 State Key Laboratory of Molecular Engineering of Polymers, State Key Laboratory of Genetic Engineering, Collaborative Innovation Center for Genetics and Development, School of Life Sciences and Human Phenome Institute, Zhangjiang Fudan International Innovation Center, Fudan University, Shanghai, China; 7 Department of Infectious Diseases, Shanghai Key Laboratory of Infectious Diseases and Biosafety Emergency Response, National Medical Center for Infectious Diseases, Huashan Hospital, Fudan University, Shanghai, China; 8 Center for Infectious Diseases, Beijing Youan Hospital, Capital Medical University, Beijing, China; 9 State Key Laboratory of Genetic Engineering, School of Life Sciences and Huashan Hospital, MOE Engineering Research Center of Gene Technology, Shanghai Engineering Research Center of Industrial Microorganisms, Fudan University, Shanghai, China; 10 Artificial Intelligence Innovation and Incubation Institute, Fudan University, Shanghai, China; 11 National Clinical Research Center for Aging and Medicine, Huashan Hospital, Fudan University, Shanghai, China

**Keywords:** Immunology, Molecular biology

Dear Editor,

Since its emergence in late 2021, the SARS-CoV-2 Omicron variant has continued to evolve and given rise to numerous subvariants. Due to the high transmissibility and immune escape properties, Omicron BA.2 caused a local outbreak in Shanghai since March 2022 and resulted in over 0.6 million laboratory-confirmed infections^1,2^. Since the “zero-Covid” policy was lifted in December 2022, China has experienced a surge in COVID-19 infections nationwide. However, the variant composition was much simpler in China, with only two main subvariants, BA.5 and BF.7, during this recent COVID-19 infection wave according to the sequences deposited in the GISAID database (Supplementary Fig. S1a).

We have previously reported that BA.1, BA.1.1, BA.2, and BA.3 sub-lineages^3,4^, as well as BA.2 descendants and BA.4/5^5^ evaded neutralizing antibodies induced by vaccination and infection. In this study, we constructed a panel of pseudoviruses (PsVs) representing Omicron subvariants circulating with high frequency recently, BA.2.75, BN.1, CH.1.1, XBB.1, XBB.1.5, BA.5, BF.7, BQ.1 and BQ.1.1, and several other with mutations of interest, such as BA.2.75.2, BA.2.75.6, CA.3.1, BA.5.2.7, BE.1.1.1, BF.14 and BF.16 (Supplementary Fig. S1b). Given the rapid growth and ever-increasing spike mutation complexity of these sub-lineages, several groups have tested the neutralization evasion by some of these subvariants^6–12^. However, a comprehensive assessment of booster vaccination or breakthrough infection sera against all of these distinct Omicron sub-lineages, especially an independent evaluation on the neutralization activity of sera from the previous (Delta and BA.2) and recent (BA.5 and BF.7) waves in China, is still crucial for the global public health.

We first collected sera from healthy adults at day 14 post homologous booster with CoronaVac, or heterologous booster with ZF2001, primed with two doses of CoronaVac (Supplementary Table S1), and tested their neutralization activity against this panel of PsVs. As shown in Fig. 1a, the booster groups had detectable neutralizing geometric mean titers (GMTs) against WT (D614G), but the GMTs for BA.2 and BA.4/5 showed 6–16-fold reductions compared to WT. And the neutralizing titers against the BA.2- and BA.5-derivative variants decreased further, with CA.3.1, CH.1.1, XBB.1 and XBB.1.5 being the strongest escaped BA.2 descendants (4–8-fold reductions compared to BA.2), and BQ.1 and BQ.1.1 being the strongest escaped BA.5 descendants (2–3-fold reductions compared to BA.5).

**Fig. 1:**
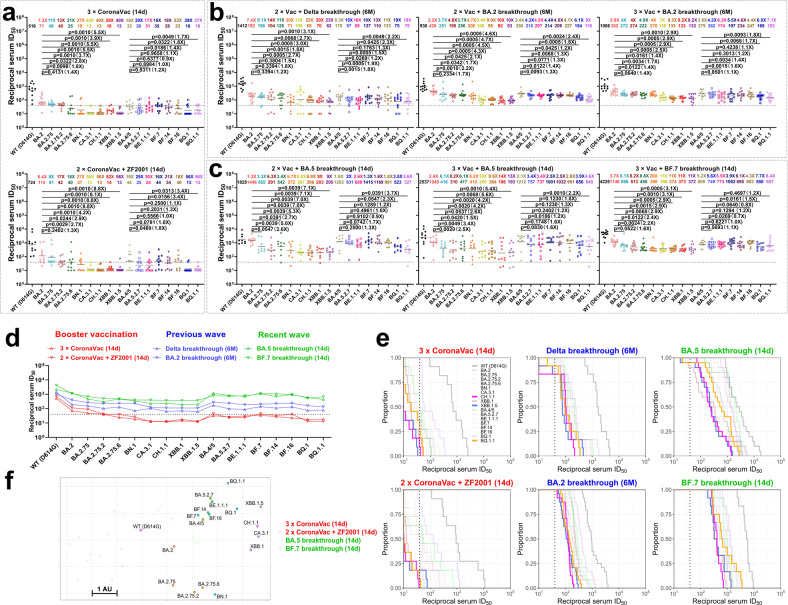
Neutralization of the Omicron subvariants by booster vaccination or breakthrough infection sera from previous or recent waves in China. Neutralization of pseudotyped WT (D614G) and Omicron sub-lineage viruses by sera collected from (**a**) individuals at day 14 after vaccinated with a CoronaVac or ZF2001 following two doses of CoronaVac; (**b**) individuals at 6 month after infection with Delta virus post two doses of inactivated vaccine, or from those infected with BA.2 virus post two or three doses of inactivated vaccine in previous waves in China; (**c**) individuals at day 14 after infection with BA.5 virus post two or three doses of inactivated vaccine, or from those infected with BF.7 virus post three doses of inactivated vaccine in recent wave in China. The neutralization titer (ID_50_) was defined as the reciprocal value of the sample dilution at which 50% neutralization was attained. **d** In parallel comparison of neutralization GMTs against distinct Omicron subvariants. **e** Cumulative distribution function plots of titers against Omicron subvariants, showing the proportion of samples at or above a given titer. **f** Antigenic map based on the day 14 serum neutralization data. Virus positions are represented by closed circles whereas serum positions are shown as open or closed squares. Both axes represent antigenic distance with one antigenic distance unit (AU) in any direction corresponding to a 2-fold change in neutralization ID_50_ titer. Values above the symbols denote GMTs and the fold-change was calculated by comparing the titer to WT. For each BA.2 or BA.5 descendant, comparison was also done with BA.2 or BA.5. Dotted lines indicate the threshold of detection (40 for all the cohorts). *P* values were determined by using Multiple Mann–Whitney tests. WT wild-type.

We previously reported that BA.2 breakthrough infection significantly increased neutralization titers against BA.2, its derivative variants and BA.4/5^5^, at day 14 post-infection. To determine whether those vaccinated individuals infected during the previous waves still maintain adequate neutralization titers against the newly emerging viruses after a longer time, we collected sera from individuals who had previously had Delta or BA.2 breakthrough infections at 6 months post-infection and examined the degree of neutralizing antibody escape. For sera from Delta breakthrough infection after two inactivated vaccination doses, we noticed a trend of neutralization titer decrease for the Omicron subvariants, similar to the booster vaccination groups, and CH.1.1, XBB.1, XBB.1.5, BQ.1 and BQ.1.1 showing the strongest serum escape, amounting to about 20-fold reductions in potency compared to WT (Fig. 1b, left panel). While for the BA.2 breakthrough infection individuals, we further divided them into two groups based on whether they had two or three inactivated vaccination doses prior to infection. In these two groups, the neutralization titers for Omicron subvariants were lower than that for WT, but the decline levels were less significant than those in the booster vaccination or Delta breakthrough infection groups. For instance, the neutralization GMTs against BA.2 dropped ~2–3-fold in BA.2 breakthrough infection groups, compared to the ~7-fold in the booster vaccination or Delta breakthrough infection groups (Fig. 1a, b). When comparing the BA.2 breakthrough infection with the Delta breakthrough infection (both groups had the same 2-dose vaccination history), the BA.2 breakthrough infection sera kept higher titers against BA.2 and BA.5, as well as some of their descendant viruses (Supplementary Fig. S2a), which may be associated with the antigenic difference between Omicron and Delta variants. Again, CA.3.1, CH.1.1, XBB.1 and XBB.1.5 were the strongest escaped BA.2 descendants (7–10-fold reductions compared to WT and 2.5–4.7-fold reductions compared to BA.2), and BQ.1 and BQ.1.1 were the strongest escaped BA.5 descendants (6–8-fold reductions compared to WT and ~2-fold reductions compared to BA.5) (Fig. 1b, middle and right panels).

We then examined the resistance of these Omicron subvariants to sera from individuals who had more recently had BA.5 or BF.7 breakthrough infections, collected at day 14 post-infection. Overall, BA.5 breakthrough infection induced high neutralizing titers for Omicron subvariants. The GMTs for BA.5 and some of its derivative variants like BF.7 were all above 1000, and even for the BQ.1 and BQ.1.1 subvariants, the GMTs were sustained above 300. Certain BA.2 descendant viruses, such as BA.2.75.2, BN.1, CA.3.1, CH.1.1, XBB.1 and XBB.1.5 exhibited stronger evasion from the BA.5 breakthrough infection sera, but they still retained GMTs ~200 or higher (Fig. 1c, left and middle panels). Moreover, similar to the BA.2 breakthrough infection, the BA.5 breakthrough infection elicited similar levels of neutralizing antibodies in vaccinated people regardless of whether they had booster (Supplementary Fig. S2b, c). For people who had received three-dose inactivated vaccines prior to BF.7 breakthrough infection, they all had relatively high neutralization activities against BA.5 and its derivative variants, including BQ.1 and BQ.1.1 (GMTs > 500), with the highest titer against BF.7 virus as expected. Although some BA.2 descendants, BN.1, CA.3.1, CH.1.1, XBB.1 and XBB.1.5 exhibited the lowest neutralization sensitivity, which might be explained by their antigenic distance from BF.7, their neutralization GMTs were all above 300 (Fig. 1c, right panel). When compared with the BA.5 breakthrough infection data, the BF.7 breakthrough infection mounted generally comparable neutralizing responses (Supplementary Figs. S2d and S3).

We also compared different cohorts in parallel. People with booster vaccination only had the lowest serum neutralization activity, with almost undetectable titers against BQ.1, BQ.1.1, CA.3.1, CH.1.1, XBB.1, and XBB.1.5. Those vaccinated and infected by Delta or BA.2 variants in previous waves still retained appreciable activity against the distinct Omicron subvariants 6 months post-infection. The highest neutralization activity came from those vaccinated and infected with BA.5 or BF.7 viruses in the recent wave in China, which could be attributed to the higher antigenic similarity of BA.5 or BF.7 with the newly emerged viruses as well as the shorter sample collection time post-infection (Fig. 1d). Large proportion of the sera from the booster vaccination-only groups had neutralizing antibody titers under our threshold of detection, especially for BQ.1, BQ.1.1, CA.3.1, CH.1.1, XBB.1, and XBB.1.5 subvariants. However, almost all sera from the Omicron breakthrough infection groups had neutralizing antibody titers above our threshold of detection, and the overall trend of curves shifting to the right further indicated the enhanced neutralization potency (Fig. 1e). Taken together, these results indicated that people vaccinated and then infected in the recent wave in China should have neutralizing activity against the main circulating SARS-CoV-2 viruses, at least for a short period of time.

To visualize and quantify the antigenic distances among WT and the Omicron subvariants, we utilized all the day 14 serum neutralization results to construct an antigenic map (Fig. 1f). While some Omicron subvariants like BA.4/5, BA.5.2.7, BE.1.1.1, BF.14, and BF.16 tend to group in a cluster, CA.3.1, CH.1.1, and the BQ and XBB subvariants have drifted away from BA.2 and BA.4/5 antigenically. Based on each antigenic unit equaling a twofold difference in virus neutralization, BQ.1.1 is ~12-fold more resistant to serum neutralization than WT, while CH.1.1 and XBB.1.5 are ~20-fold more resistant to serum neutralization than WT, raising concerns about how the antigenic drift will affect vaccine efficacy in the real world. Continued vigilance and sustained support for development of updated vaccine that protect broadly are of great importance.

## Supplementary information

Supplementary Information
