# Supplementary material for: Neutralization of SARS-CoV-2 BQ.1.1, CH.1.1, and XBB.1.5 by breakthrough infection sera from previous and recent waves in China
Source: Cell Discov. 2023 Jun 27;9:64. doi: 10.1038/s41421-023-00569-5 (PMC10300020; doi:10.1038/s41421-023-00569-5)
Supplement: Supplementary file 1 — Supplementary Information [file 41421_2023_569_MOESM1_ESM.pdf]

Supplementary Information

Neutralization of SARS-CoV-2 BQ.1.1, CH.1.1, and XBB.1.5 by Breakthrough Infection Sera from Previous and Recent Waves in China

Xun Wang[1,2,#], Shuai Jiang[1,3,#], Shujun Jiang[4,5,#], Xiangnan Li[6,#], Jingwen Ai[2,7,#], Ke Lin[2,7,#], Shiyun Lv[8,#], Shixuan Zhang[6,#], Minghui Li[1,#], Jixi Li[9,*], Lili Dai[8,*], Zixin Hu[1,6,10,*], Wenhong Zhang[2,7,11,*], Yanliang Zhang[4,5,*], Pengfei Wang[1,2,*]

[1]Shanghai Pudong Hospital, Fudan University Pudong Medical Center, Shanghai Institute of Infectious Disease and Biosecurity, State Key Laboratory of Genetic Engineering, MOE Engineering Research Center of Gene Technology, School of Life Sciences, Fudan University, Shanghai, China

[2]Shanghai Huashen Institute of Microbes and Infections, Shanghai, China

[3]Department of General Surgery, Shanghai Pudong Hospital, Fudan University Pudong Medical Center, Shanghai Key Laboratory of Vascular Lesions Regulation and Remodeling, Shanghai, China.

[4]Department of Infectious Diseases, Nanjing Hospital of Chinese Medicine Affiliated to Nanjing University of Chinese Medicine, Nanjing, Jiangsu, China

[5]Nanjing Research Center for Infectious Diseases of Integrated Traditional Chinese and Western Medicine, Nanjing, Jiangsu, China

[6]State Key Laboratory of Molecular Engineering of Polymers, State Key Laboratory of Genetic Engineering, Collaborative Innovation Center for Genetics and Development, School of Life Sciences and Human Phenome Institute, Zhangjiang Fudan International Innovation Center, Fudan University, Shanghai, China

[7]Department of Infectious Diseases, Shanghai Key Laboratory of Infectious Diseases and Biosafety Emergency Response, National Medical Center for Infectious Diseases, Huashan Hospital, Fudan University, Shanghai, China

[8]Center for Infectious Diseases, Beijing Youan Hospital, Capital Medical University, Beijing, China

[9]State Key Laboratory of Genetic Engineering, School of Life Sciences and Huashan Hospital, MOE Engineering Research Center of Gene Technology, Shanghai Engineering Research Center of Industrial Microorganisms, Fudan University, Shanghai, China

[10]Artificial Intelligence Innovation and Incubation Institute, Fudan University, Shanghai, China

[11]National Clinical Research Center for Aging and Medicine, Huashan Hospital, Fudan University, Shanghai, China

[#]These authors contributed equally

[*]Address correspondence to Pengfei Wang (pengfei_wang@fudan.edu.cn), Yanliang Zhang (fsyy00404@njucm.edu.cn), Wenhong Zhang (zhangwenhong@fudan.edu.cn), Zixin Hu (huzixin@fudan.edu.cn), Lili Dai (lilydaier@ccmu.edu.cn) or Jixi Li (lijixi@fudan.edu.cn).

Methods

Serum samples

Sera from individuals who received two or three doses of inactivated vaccine (CoronaVac) or recombinant protein subunit (ZF2001) vaccine were collected at Nanjing Hospital of Chinese Medicine 14 days after the final dose. 12 individuals who were breakthrough infected with SARS-CoV-2 Delta (B.1.617.2) variant after receiving two doses of inactivated vaccine were recruited at the Nanjing Hospital of Chinese Medicine. 24 individuals who were infected with BA.2 variant after receiving two or three doses of inactivated vaccine were recruited at Huashan Hospital, Fudan University. 20 individuals who were infected with BA.5 variant after receiving two or three doses of inactivated vaccine were recruited at the Nanjing Hospital of Chinese Medicine. 12 individuals who were infected with SARS-CoV-2 BF.7 variant after receiving three doses of inactivated vaccine were recruited at the Youan Hospital, Capital Medical University. For all COVID-19 participants, the clinical diagnosis criteria were based on the ninth National COVID-19 guidelines. The SARS-CoV-2 infection of all the subject was confirmed by Polymerase Chain Reaction (PCR) and sequencing. All participants involved in this study had mild symptoms. Their baseline characteristics are summarized in Table S1. All the participants provided written informed consents. All collections were conducted according to the guidelines of the Declaration of Helsinki and approved by the ethical committee of Huashan Hospital Affiliated to Fudan University (number KY2022-596 & KY2021-749) and Nanjing Hospital of Chinese Medicine Affiliated to Nanjing University of Chinese Medicine (number KY2021162).

Cell lines

Expi293F cells (Thermo Fisher Cat# A14527) were cultured in the serum free SMM 293-TI medium (Sino Biological Inc.) at 37 °C with 8% CO_2 on an orbital shaker platform. HEK293T cells (Cat# CRL-3216), Vero E6 cells (cat# CRL-1586) were from ATCC and cultured in 10% Fetal Bovine Serum (FBS, GIBCO cat# 16140071) supplemented Dulbecco's Modified Eagle Medium (DMEM, ATCC cat# 30-2002) at 37°C, 5% CO_2. I1 mouse hybridoma cells (ATCC, cat# CRL-2700) were cultured in Eagle's Minimum Essential Medium (EMEM, ATCC cat# 30-2003)) with 20% FBS.

Construction and production of variant pseudoviruses

Plasmids encoding the WT (D614G) SARS-CoV-2 spike and Omicron sub-lineage spikes were constructed. HEK293T cells were transfection with the indicated spike gene using Polyethylenimine (Polyscience). Cells were cultured overnight at 37°C with 5% CO_2 and VSV-G pseudo-typed ΔG-luciferase (G*ΔG-luciferase, Kerafast) was used to infect the cells in DMEM at a multiplicity of infection of 5 for 4 h before washing the cells with 1×DPBS three times. The next day, the transfection supernatant was collected and clarified by centrifugation at 3000g for 10 min. Each viral stock was then incubated with 20% I1 hybridoma (anti-VSV-G; ATCC, CRL-2700) supernatant for 1 h at 37 °C to neutralize the contaminating VSV-G pseudotyped ΔG-luciferase virus before measuring titers and making aliquots to be stored at −80 °C.

Pseudovirus neutralization assays

Neutralization assays were performed by incubating pseudoviruses with serial dilutions of monoclonal antibodies or sera, and scored by the reduction in luciferase gene expression. In brief, Vero E6 cells were seeded in a 96-well plate at a concentration of 2×10^4 cells per well. Pseudoviruses were incubated the next day with serial dilutions of the test samples in triplicate for 30 min at 37 °C. The mixture was added to cultured cells and incubated for an additional 24 h. The luminescence was measured by Luciferase Assay System (Beyotime). ID_{50} was defined as the dilution at which the relative light units were reduced by 50% compared with the virus control wells (virus + cells) after subtraction of the background in the control groups with cells only. The ID_{50} values were calculated using nonlinear regression in GraphPad Prism.

Antigenic Cartography

The constructed antigenic map was based on serum neutralization data utilizing the antigenic cartography methods[1,2], which are implemented in the Racmacs package (https://acorg.github.io/Racmacs/). The antigenic map was generated in R with 10000 optimization steps and other default parameters in a 2-dimesional space. The distances between positions of sub-lineages and serum on the antigenic map were optimized so that distances approach the fold decreases in neutralizing ID_{50} titer, relative to the maximum titer for each serum. Each unit of distance in arbitrary directions in the antigenic map represents a 2-fold change in the ID_{50} titer.

ID_{50} Cumulative Distribution Analysis

The ID_{50} cumulative distributions of different sub-lineages were determined by the proportion of samples at or above a given titer at different vaccination/infection statuses. The max ID_{50} on the Cumulative Distribution figure were assumed as the 1.1-fold of the maximum titer for each sub-lineage.

Prevalence of Different Sub-lineages

The prevalence of different Omicron sub-lineages were summarized using Embers in COVID-19 Viral Genomic Analysis pipeline[3] (https://cov.lanl.gov/content/index), whose data were from GISAID. The prevalence data for Global and China were collected from Dec 1st, 2021 to Mar 15th, 2023 and summarized by “Grouped Pango lineages”.

Sequence alignment and phylogenetic tree construction

This analysis involved 320 nucleotide sequences, including 20 samples for each lineage (Wuhan, Alpha, Beta, Gamma, Delta, BA.1, BA.2, BA.4, BA.5, BA.2.75, BN.1, BF.7, BQ.1, BQ.1.1, XBB.1, XBB.1.5, CA.3.1 and CH.1.1) randomly selected from GISAID database. Sample set can be found at https://github.com/wenrurumon/GISAID/blob/main/2022.04.07.487489/map2.csv. Sequence alignment was carried out using MAFFT progress[4] and corrected manually. The evolutionary history was inferred using the Neighbor-Joining method. In order to present the evolutionary relationship more accurately and finely, we adjusted the samples. There was a total of 155 sequences in the final dataset. The optimal tree was shown. Evolutionary analysis was conducted in FastTree[5] and visualized in iTOL v6.

Quantitative and statistical analysis

The statistical analyses for the pseudovirus virus neutralization assessments were performed using GraphPad Prism for calculation of mean value for each data point. Each specimen was tested in triplicate. Antibody neutralization ID_{50} values were calculated using a five-parameter dose-response curve in GraphPad Prism. For comparing the serum neutralization titers, statistical analysis was performed using Multiple Mann-Whitney tests. Two-tailed p values are reported. No statistical methods were used to determine whether the data met assumptions of the statistical approach.

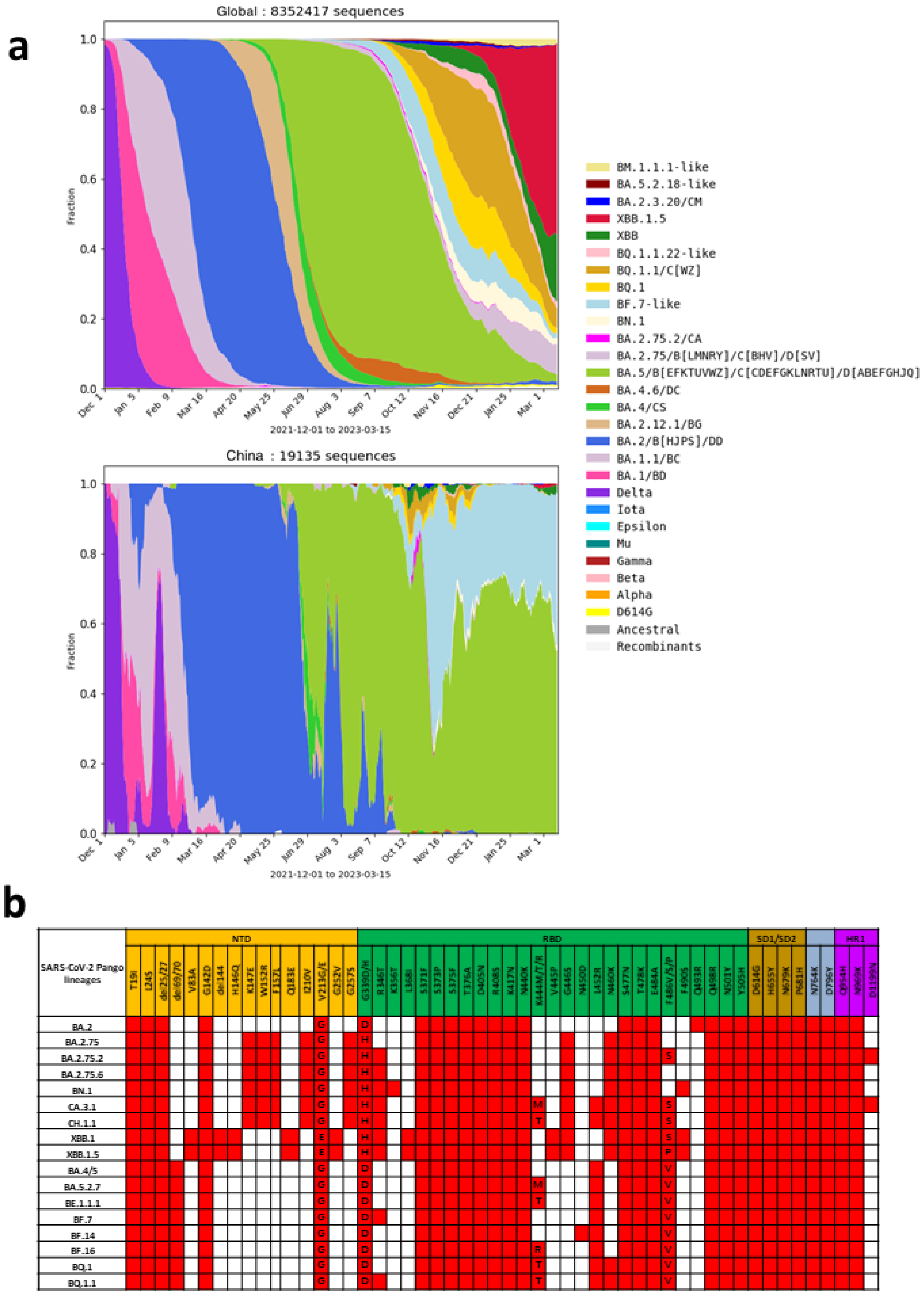

Supplementary Fig. S1. Characteristics of the Omicron subvariants.

(a) Prevalence of the Omicron subvariants based on all the sequences from the globe or China available on GISAID from Dec 1st, 2021 to Mar 15th, 2023.

(b) Spike mutations within the Omicron subvariants.

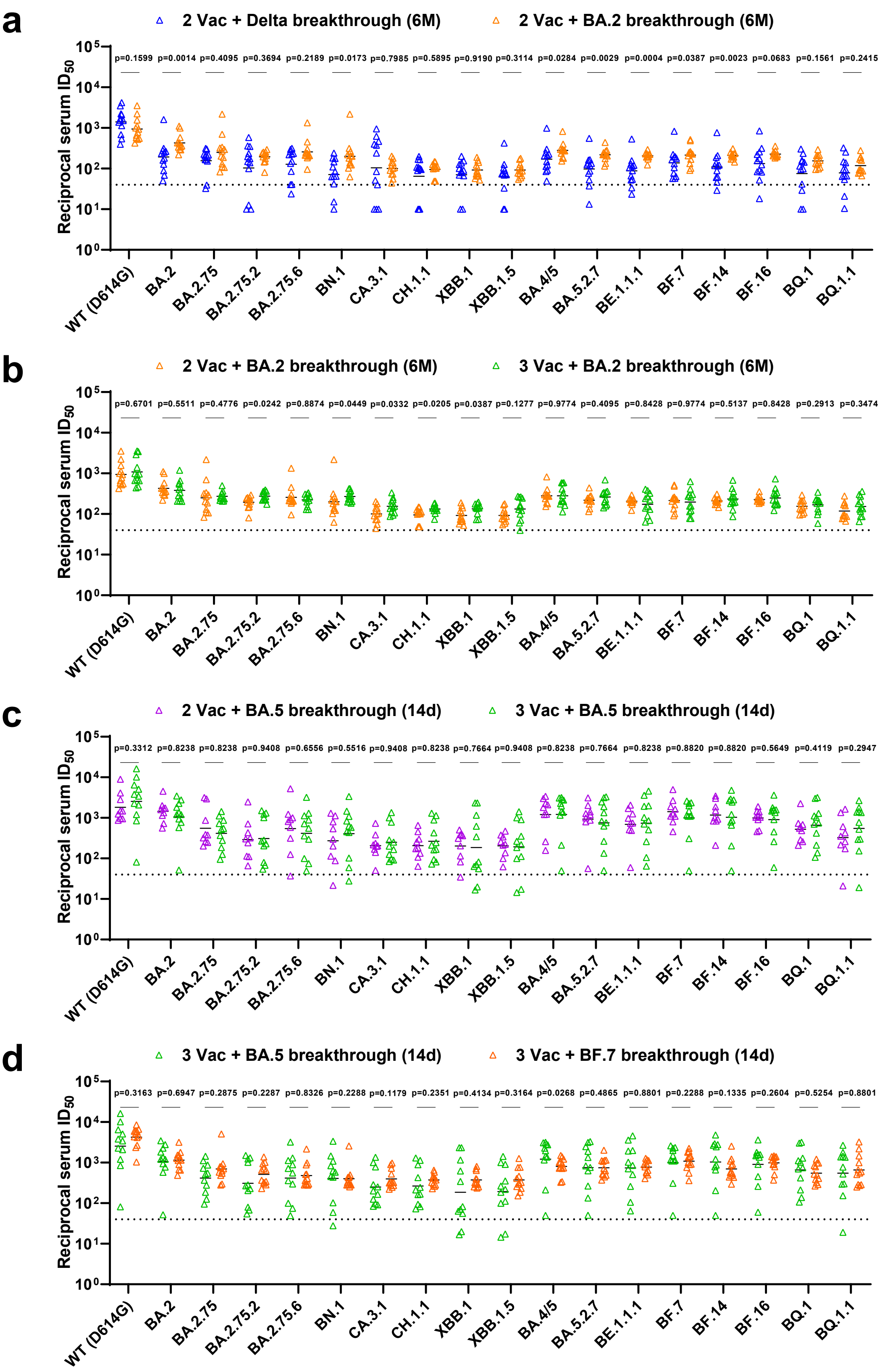

Supplementary Fig. S2. In parallel comparison of serum neutralization titers against distinct SARS-CoV-2 variants. (a) Neutralization titers of sera collected at month 6 from individuals with Delta breakthrough infection versus those with BA.2 breakthrough infection after 2 doses of

inactivated vaccinations. (b) Neutralization titers of sera collected at month 6 from individuals with BA.2 breakthrough infection after 2 doses versus those after 3 doses of inactivated vaccinations. (c) Neutralization titers of sera collected at day 14 from individuals with BA.5 breakthrough infection after 2 doses versus those after 3 doses of inactivated vaccinations. (d) Neutralization titers of sera collected at day 14 from individuals with BA.5 breakthrough infection versus those with BF.7 breakthrough infection after 3 doses of inactivated vaccinations. Dotted lines indicate the threshold of detection. *P* values were determined by multiple Mann-Whitney tests.

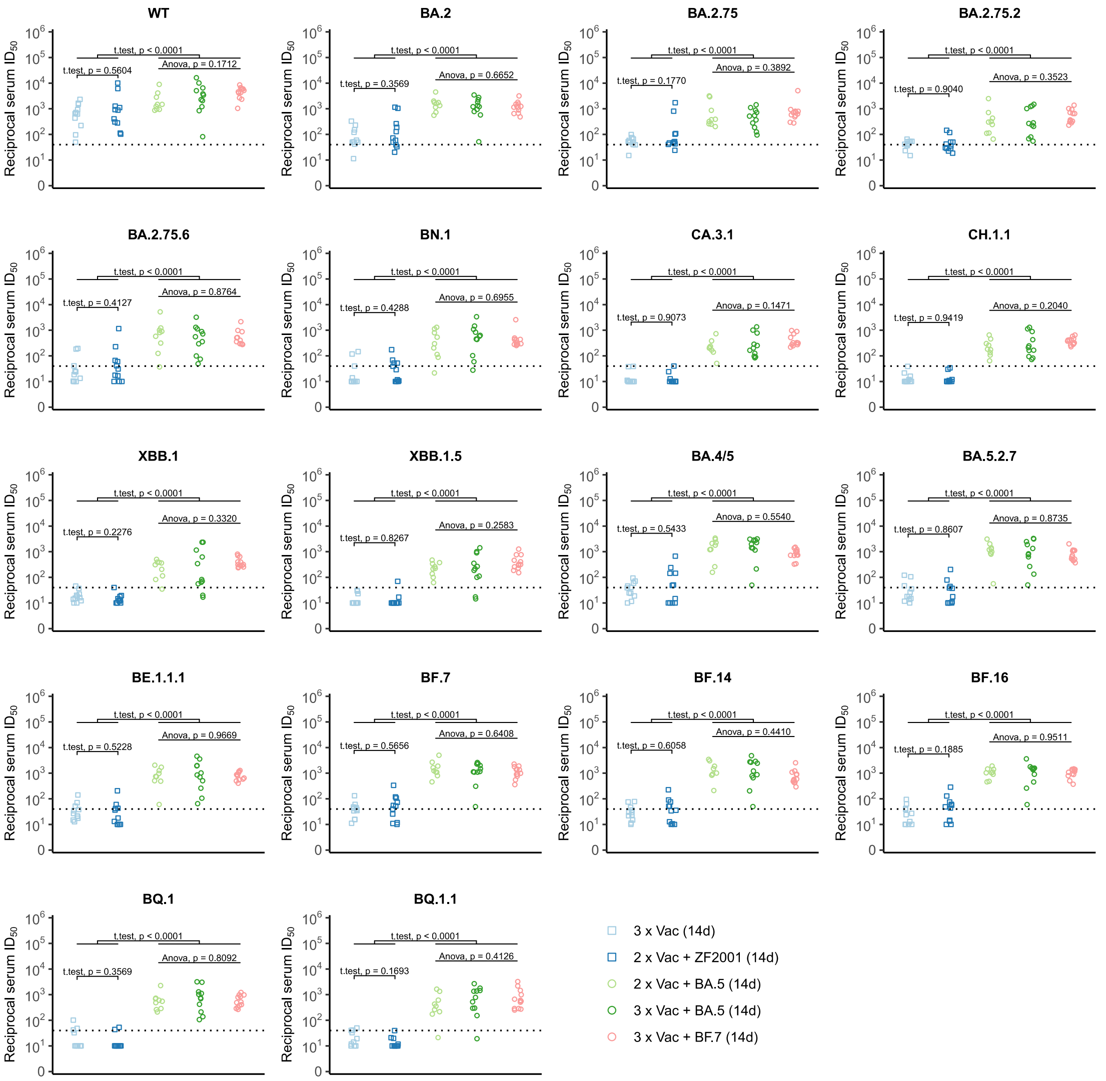

Supplementary Fig. S3. In parallel comparison of serum neutralization titers at day 14 from vaccinations or Omicron breakthrough infection. Neutralization titers of sera collected at day 14 from individuals with homologous or heterologous booster doses, or from individuals with BA.5 breakthrough infection after 2 or 3 doses of inactivated vaccinations, or from individuals with BF.7 breakthrough infection after 3 doses of inactivated vaccinations. Dotted lines indicate the threshold of detection. *P* values were determined by t test or one-way ANOVA as indicated.

Supplementary Table S1. Baseline characteristics of enrolled participants

	3 × CoronaVac (n=11)	2 × CoronaVac + ZF2001 (n=11)	2 × Vac + Delta breakthrough (n=12)	2 × Vac + BA.2 breakthrough (n=12)	3 × Vac + BA.2 breakthrough (n=12)	2 × Vac + BA.5 breakthrough (n=9)	3 × Vac + BA.5 breakthrough (n=11)	3 × Vac + BF.7 breakthrough (n=12)
Age(years), median (range)	44.3(28-63)	39.7(20-53)	47(35-55)	43(25-66)	37(32-66)	30.8(23-47)	30.6(22-47)	40.1 (34-50)
Male, n (%)	7(63.6%)	3(27.2%)	5(41.6%)	9 (75.0%)	8(66.7%)	6(66.6%)	5(45.5%)	4(33.3%)
BMI (kg/m^2), mean (SD)	24.7(3.1)	23.9(3.7)	29(10.7)	25.1(2.6)	25.2(3.2)	24(3.2)	20.9(2.2)	23.9(3.3)
Breakthrough infections days after the last Coronavirus vaccines, median (range)	N/A	N/A	76.4 (34-128)	ND	ND	458.3 (325-587)	313.5 (220-387)	407.4 (363-432)
Serum samples collection days after the vaccination or infection, median (range)	17.5（15-22）	18（18）	156.6（153-161）	180(164-202)	176(167-196)	25.2（21-34）	21.9（18-24）	14.1（10-19）
Comorbidities (%)								
Any, n (%)	0(0%)	0(0%)	2(16.6%)	3(25%)	0(0%)	0(0%)	0(0%)	0(0%)
HTN, n (%)	0(0%)	0(0%)	2(16.6%)	3(25%)	0(0%)	0(0%)	0(0%)	0(0%)
CAD, n (%)	0(0%)	0(0%)	0(0%)	0(0%)	0(0%)	0(0%)	0(0%)	0(0%)
DM, n (%)	0(0%)	0(0%)	0(0%)	0(0%)	0(0%)	0(0%)	0(0%)	0(0%)
NASH, n (%)	0(0%)	0(0%)	0(0%)	0(0%)	0(0%)	0(0%)	0(0%)	0(0%)
Arrhy, n (%)	0(0%)	0(0%)	0(0%)	0(0%)	0(0%)	0(0%)	0(0%)	0(0%)
Asthma, n (%)	0(0%)	0(0%)	0(0%)	0(0%)	0(0%)	0(0%)	0(0%)	0(0%)
Rhinitis, n (%)	0(0%)	0(0%)	0(0%)	0(0%)	0(0%)	0(0%)	0(0%)	0(0%)
Urticaria, n (%)	0(0%)	0(0%)	0(0%)	0(0%)	0(0%)	0(0%)	0(0%)	0(0%)

N/A, not applicable. ND, no data. BMI, body mass index. CAD, coronary artery disease. HTN, hypertension. DM, diabetes mellitus. Arrhy, arrhythmia, NASH, non-alcoholic steatohepatitis.

REFERENCES

1 Smith, D. J. *et al.* Mapping the Antigenic and Genetic Evolution of Influenza Virus. *Science* **305**, 371-376 (2004). https://doi.org:10.1126/science.1097211

2 Wilks, S. H. *et al.* Mapping SARS-CoV-2 antigenic relationships and serological responses. *bioRxiv* (2022). https://doi.org:10.1101/2022.01.28.477987

3 Korber, B. *et al.* Tracking Changes in SARS-CoV-2 Spike: Evidence that D614G Increases Infectivity of the COVID-19 Virus. *Cell* **182**, 812-827 e819 (2020). https://doi.org:10.1016/j.cell.2020.06.043

4 Katoh, K., Misawa, K., Kuma, K. i. & Miyata, T. MAFFT: a novel method for rapid multiple sequence alignment based on fast Fourier transform. *Nucleic Acids Research* **30**, 3059-3066 (2002). https://doi.org:10.1093/nar/gkf436

5 Price, M. N., Dehal, P. S. & Arkin, A. P. FastTree: Computing Large Minimum Evolution Trees with Profiles instead of a Distance Matrix. *Molecular Biology and Evolution* **26**, 1641-1650 (2009). https://doi.org:10.1093/molbev/msp077
